# Supplementary material for: Structural and functional analysis of VYD222: a broadly neutralizing antibody against SARS-CoV-2 variants
Source: bioRxiv. 2025 Aug 28:2025.08.28.672883. Preprint. [Version 1] doi: 10.1101/2025.08.28.672883 (PMC12407960; doi:10.1101/2025.08.28.672883)
Supplement: Supplement 1 [file media-1.pdf]

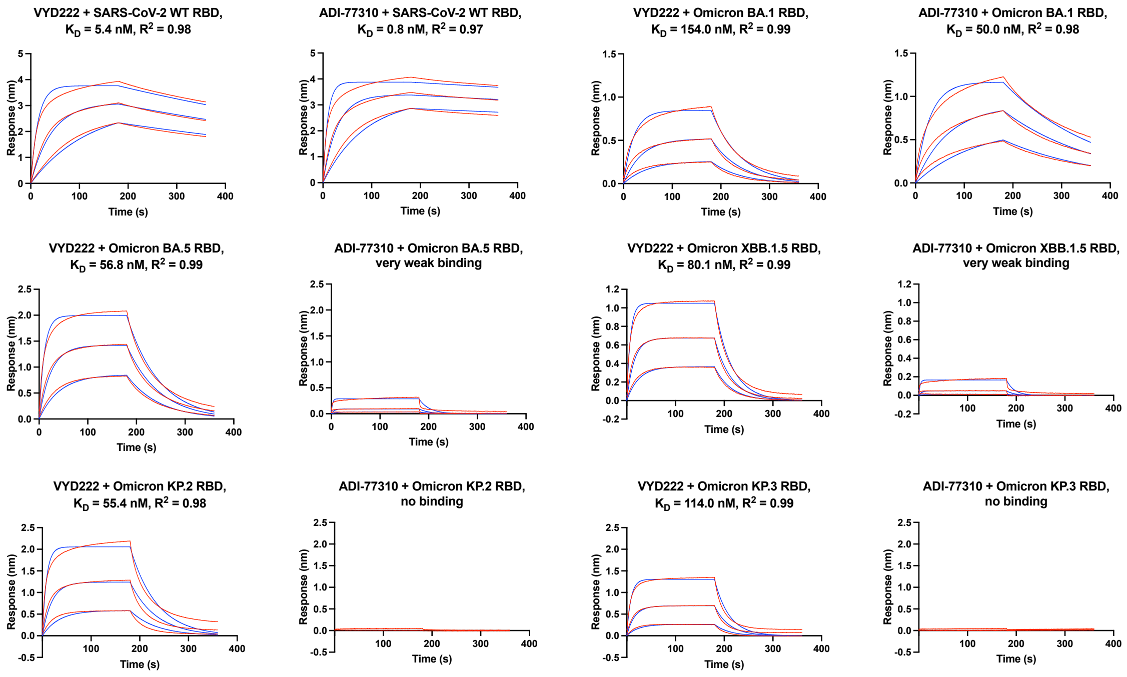

Figure S1. Sensorgrams for binding of Fabs to RBDs of SARS-CoV-2 measured by biolayer interferometry (BLI). X-axis represents time of reaction (s). Y-axis represents the response. Blue lines represent the response curves and red lines represent the 1:1 binding model. Binding kinetics were measured for the RBDs at 5-fold dilution ranging from 500 nM to 20 nM.

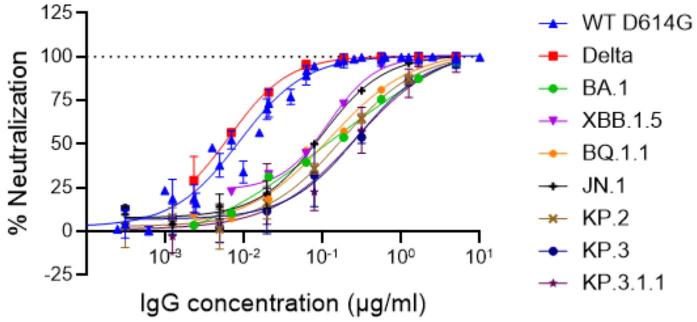

Figure S2. Dose-response curves in pseudovirus neutralization assay for VYD222. Percent neutralization values obtained from the PhenoSense assay for VYD222 against the indicated variant were curve fitted using a 4PL nonlinear regression model (GraphPad Prism version 10.1.2).

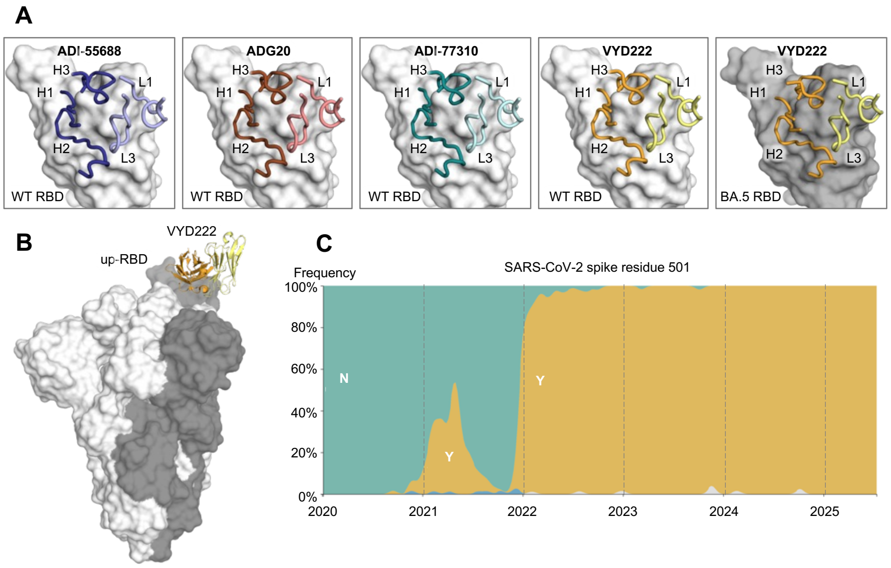

Figure S3. Interactions between SARS-CoV-2 spike and ADI-55688, ADG20, ADI-77310, and VYD222. (A) WT and BA.5 RBDs are represented by white and dark grey surfaces. CDRs that interact with SARS-CoV-2 are shown as tubes. **(B)** VYD222/RBD complex structure superimposed onto a full spike protein (PDB 8G71). The protomers with RBDs in down conformations are in white, while that with an RBD in the up position is in dark grey. **(C)** Frequency of amino acids at position 501 on SARS-CoV-2 spike as of July 2025. Data were sourced from nextstrain.org (GISAID data) (*1, 2*).

A
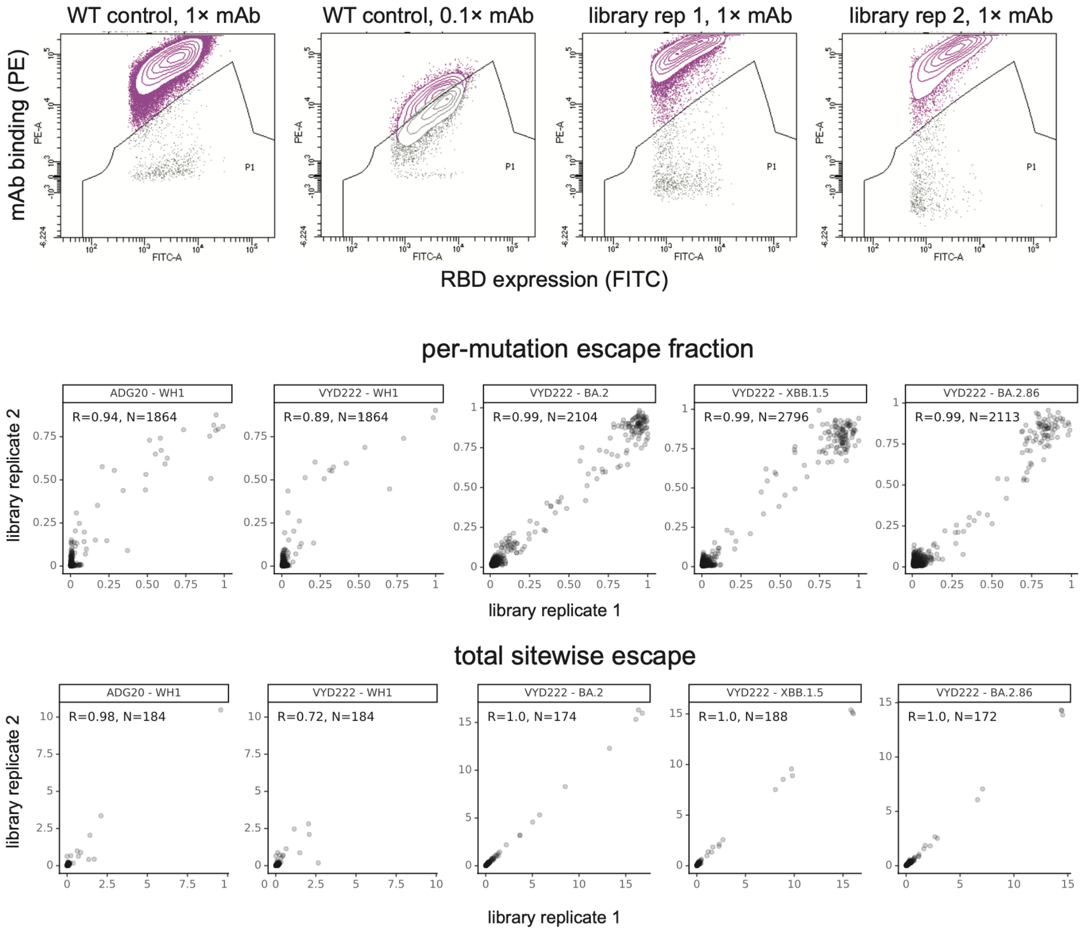

Figure S4. ADG20 or VYD222 binding to SARS-CoV-2 variant deep mutational scanning libraries. (A) Representative FACS gates used to identify mutations that escape antibody binding. An antibody-escape gate is drawn that captures approximately 50% of the cells in the respective wildtype control labeled at 0.1x the library selection antibody concentration. The "escape fraction" determined from deep sequencing of pre- and post-sort populations estimates the fraction of yeast cells expressing a particular mutant genotype that fell into this antibody-escape FACS gate. **(B)** For each experiment, the correlation in the per-mutation escape fraction (top) or total escape per site (sum of per-mutation escape fractions; bottom) between duplicate library selections.

	403	404	405	406	408	409	436	437	439	493	500	501	502	503	504	505	506
SARS-CoV-2_MN908947	R	G	D	V	R	Q	W	N	N	Q	T	N	G	V	G	Y	Q
alpha	R	G	D	V	R	Q	W	N	N	Q	T	Y	G	V	G	Y	Q
beta	R	G	D	V	R	Q	W	N	N	Q	T	Y	G	V	G	Y	Q
gamma	R	G	D	V	R	Q	W	N	N	Q	T	Y	G	V	G	Y	Q
mu	R	G	D	V	R	Q	W	N	N	Q	T	Y	G	V	G	Y	Q
delta	R	G	D	V	R	Q	W	N	N	Q	T	N	G	V	G	Y	Q
KP.3.1.1	K	G	N	V	S	Q	W	N	N	E	T	Y	G	V	G	H	Q
KP.1.1	K	G	N	V	S	Q	W	N	N	Q	T	Y	G	V	G	H	Q
KP.2	K	G	N	V	S	Q	W	N	N	Q	T	Y	G	V	G	H	Q
JN.1	K	G	N	V	S	Q	W	N	N	Q	T	Y	G	V	G	H	Q
BA.2.86	K	G	N	V	S	Q	W	N	N	Q	T	Y	G	V	G	H	Q
EG.5.1	R	G	N	V	S	Q	W	N	N	Q	T	Y	G	V	G	H	Q
XBB.1.5	R	G	N	V	S	Q	W	N	N	Q	T	Y	G	V	G	H	Q
XBB.1.16	R	G	N	V	S	Q	W	N	N	Q	T	Y	G	V	G	H	Q
BA.1	R	G	D	V	R	Q	W	N	N	R	T	Y	G	V	G	H	Q
BQ.1.1	R	G	N	V	S	Q	W	N	N	Q	T	Y	G	V	G	H	Q
BA.2	R	G	N	V	S	Q	W	N	N	R	T	Y	G	V	G	H	Q
BA.4/5	R	G	N	V	S	Q	W	N	N	Q	T	Y	G	V	G	H	Q
Rs4084_KY417144	K	G	D	V	R	Q	W	N	N	R	T	A	G	V	G	H	Q
Rs4231_KY417146	K	G	D	V	R	Q	W	N	N	R	T	A	G	V	G	H	Q
RsSHC014_KC881005	K	G	D	V	R	Q	W	N	N	R	T	A	G	V	G	H	Q
LYRa11_KF569996	K	G	D	V	R	Q	W	N	R	N	T	N	G	I	G	Y	Q
WIV16_KT444582	K	G	D	V	R	Q	W	N	R	N	T	N	G	I	G	Y	Q
WIV1_KF367457	K	G	D	V	R	Q	W	N	R	N	T	N	G	I	G	Y	Q
Rs7327_KY417151	K	G	D	V	R	Q	W	N	R	N	T	N	G	I	G	Y	Q
SARSCoV1_Sin852_HP03L_AY559082	K	G	D	V	R	Q	W	N	R	N	T	T	G	I	G	Y	Q
SARSCoV1_PC413_PC04_AY613948	K	G	D	V	R	Q	W	N	R	R	T	T	G	I	G	Y	Q
SARSCoV1_SZ3_PC03_AY304486	K	G	D	V	R	Q	W	N	R	K	T	T	G	I	G	Y	Q
Pangolin_GXP2V_EPI_ISL_410542	K	G	D	V	R	Q	W	N	V	E	T	T	G	V	N	Y	Q
RaTG13_MN996532	T	G	D	V	R	Q	W	N	K	Y	T	D	G	V	G	H	Q
Pangolin_GDconsensus_Lam2020	R	G	D	V	R	Q	W	N	N	Q	T	N	G	V	G	Y	Q
BtKY72_KY352407	K	G	D	V	R	Q	W	N	N	K	T	V	G	V	G	Y	Q

Figure S5. Sequence alignment of VYD222 epitope residues in SARS-CoV-2 variants and other sarbecoviruses. ACE2-binding sarbecoviruses were identified in Starr et al. 2022 (*3*) and updated with recently emerged SARS-CoV-2 variants.

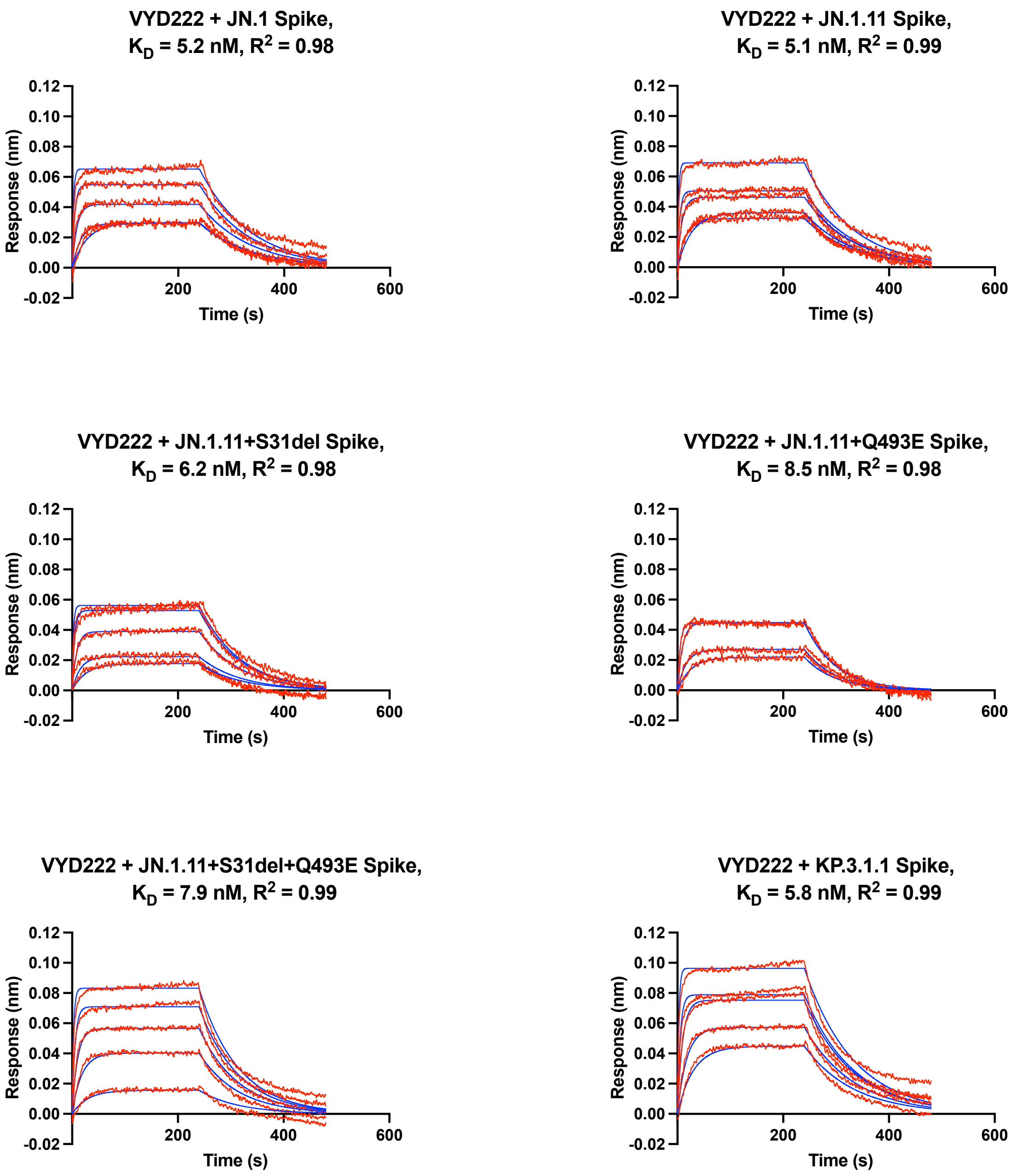

Figure S6. Sensorgrams for binding of Fabs to SARS-CoV-2 spikes measured by BLI. X-axis represents time of reaction (s). Y-axis represents the response. Blue lines represent the response curves and purple lines represent the 1:1 binding model. Binding kinetics were measured for the Fabs at 2-fold dilution ranging from 200 nM to 12.5 nM.

Table S1. X-ray data collection and refinement statistics

**Data collection**	ADI-77310 + SARS-CoV-2 RBD WT	VYD222 + SARS-CoV-2 RBD WT	VYD222 + SARS-CoV-2 RBD BA.5
Beamline	ALS5.0.1	ALS5.0.1	APS23ID-B
Wavelength (Å)	0.9774	0.9774	1.033
Space group	$P\ 4_1$	$P\ 4_1$	$P\ 4_1$
Unit cell parameters			
a, b, c (Å)	100.3, 100.3, 79.8	100.0, 100.0, 78.9	142.4, 142.4, 81.4
α, β, γ (°)	90, 90, 90	90, 90, 90	90, 90, 90
Resolution (Å) [a]	50.0-3.06	50.0-3.00	71.2-2.70
Unique reflections [a]	15,127 (722)	15,708 (774)	44,976 (2,237)
Redundancy [a]	13.4 (11.8)	13.2 (11.2)	11.8 (12.0)
Completeness (%) [a]	100 (99.9)	99.9 (99.1)	100 (99.1)
$<I/\sigma_I>$ [a]	9.3 (0.8)	11.0 (0.9)	11.4 (0.7)
R_{sym}[b] (%) [a]	27.5 (>100)	19.5 (>100)	15.9 (>100)
R_{pim}[b] (%) [a]	7.8 (64.8)	5.5 (46.6)	4.8 (48.2)
$CC_{1/2}$[c] (%) [a]	99.0 (45.8)	99.7 (61.4)	99.4 (77.6)
**Refinement statistics**			
Resolution (Å)	44.9-3.06	50.0-3.00	63.7-2.70
Reflections (work)	14,677	15,386	44,910
Reflections (test)	708	1,311	2,006
R_{cryst}[d] / R_{free}[e] (%)	23.4/26.3	22.1/26.3	20.1/25.0
No. of copies per ASU	1	1	2
No. of atoms	4,852	4,813	9,722
RBD	1,560	1,550	3,129
Fab	3,246	3,234	6,476
Ligands [f]	30	15	51
Solvent	16	14	66
Average *B*-values (Å^2)	59	67	77
RBD	58	65	96
Fab	60	68	67
Ligands	77	89	106
Solvent	48	41	59
Wilson *B*-value (Å^2)	72	71	67
**RMSD from ideal geometry**			
Bond length (Å)	0.004	0.003	0.005
Bond angle (°)	0.56	0.53	0.70
**Ramachandran statistics (%)** [g]			
Favored	95.5	95.5	94.9
Outliers	0.16	0.16	0.16
**PDB code**	9PMU	9PMV	9PMX

[a] Numbers in parentheses refer to the highest resolution shell.
[b] $R_{sym} = \Sigma_{hkl} \Sigma_i \mid I_{hkl,i} - <I_{hkl}> \mid / \Sigma_{hkl} \Sigma_i I_{hkl,i}$ and $R_{pim} = \Sigma_{hkl} (1/(n-1))^{1/2} \Sigma_i \mid I_{hkl,i} - <I_{hkl}> \mid / \Sigma_{hkl} \Sigma_i I_{hkl,i}$, where $I_{hkl,i}$ is the scaled intensity of the ith measurement of reflection h, k, l, $<I_{hkl}>$ is the average intensity for that reflection, and *n* is the redundancy.
[c] $CC_{1/2}$ = Pearson correlation coefficient between two random half datasets.
[d] $R_{cryst} = \Sigma_{hkl} \mid F_o - F_c \mid / \Sigma_{hkl} \mid F_o \mid \times 100$, where F_o and F_c are the observed and calculated structure factors, respectively.
[e] R_{free} was calculated as for R_{cryst}, but on a test set comprising 5%–8.5% of the data excluded from refinement.
[f] Bound ligands are SO_4 and acetate.
[g] From MolProbity (*4*).

Table S2. BSA values of published antibodies targeting SARS-CoV-2 RBD as calculated by PISA (5)

Name	PDB	Epitope	BSA (Å²)
CR3022	6W41	RBD	1024
CC12.1	6XC2	RBD	1347
CC12.3	6XC4	RBD	885
CV30	6XE1	RBD	1036
Fab2-4	6XEY	RBD	754
CV07-270	6XKP	RBD	817
CV07-250	6XKQ	RBD	921
HbnC3t1p1_C6	7B0B	RBD	616
COVOX-316	7BEH	RBD	740
COVOX-150	7BEI	RBD	1241
COVOX-158	7BEJ	RBD	1131
COVOX-269	7BEM	RBD	1349
COVOX-253	7BEN	RBD	749
COVOX-384	7BEP	RBD	738
ION-300	7BNV	RBD	676
P2B-2F6	7BWJ	RBD	649
B38	7BZ5	RBD	1222
CB6	7C01	RBD	1087
P2C-1F11	7CDI	RBD	958
P2C-1A3	7CDJ	RBD	922
BD-604	7CH4	RBD	1150
BD-629	7CH5	RBD	1057
BD-236	7CHB	RBD	1135
BD-368-2	7CHC	RBD	970
P5A-1D2	7CHO	RBD	1013
P5A-3C8	7CHP	RBD	1273
P22A-1D1	7CHS	RBD	1141
P4A1	7CJF	RBD	1189
P2B-1A1	7CZP	RBD	1117
P2B-1A10	7CZQ	RBD	1351
P5A-1B8	7CZR	RBD	1006
P5A-2G9	7CZT	RBD	1234
P5A-1B6	7CZU	RBD	960
P5A-2G7	7CZW	RBD	745
P5A-1B9	7CZX	RBD	890
P5A-2F11	7CZY	RBD	722
P5A-3A1	7D0C	RBD	860
Ab_58G6	7E3L	RBD	689
BD-623	7E7Y	RBD	634
BD-503	7EK0	RBD	1178
GW01	7EPX	RBD	825
BD-813	7EY0	RBD	920
BD-667	7EY4	RBD	1204
BD-821	7EY5	RBD	592
BD-804	7EYA	RBD	1085
JS026	7F7E	RBD	716
PD36-5D2	7FAE	RBD	798
T6	7FJO	RBD	718
COVA2-04	7JMO	RBD	1191
COVA2-39	7JMP	RBD	672
COVA1-16	7JMW	RBD	827
S2H13	7JV2	RBD	701
S2A4	7JVA	RBD	856
S2H14	7JX3	RBD	1070
S2-M11	7K43	RBD	657
C102	7K8M	RBD	1069
C002	7K8S	RBD	801
C119	7K8W	RBD	801
C144	7K90	RBD	800
C1A-B3	7KFV	RBD	1206
C1A-B12	7KFW	RBD	1123
C1A-C2	7KFX	RBD	1171
C1A-F10	7KFY	RBD	1141
Bamlanivimab	7KMG	RBD	879
LY-CoV488	7KMH	RBD	903
LY-CoV481	7KMI	RBD	1126
Fab2-43	7L56	RBD	755
Fab2-15	7L5B	RBD	893
AZD-8895	7L7D	RBD	647
AZD-1061	7L7E	RBD	757
DH1041	7LAA	RBD	721
DH1047	7LD1	RBD	806
DH1043	7LJR	RBD	1089
CV38-142	7LM8	RBD	862
CV05-163	7LOP	RBD	1045
CV503	7LQ7	RBD	1054
Fab1-57	7LS9	RBD	784
Fab2-7	7LSS	RBD	768
CV2-75	7M3I	RBD	809
BG4-25	7M6D	RBD	1120
BG10-19	7M6E	RBD	1018
BG7-15	7M6G	RBD	755
Zhou_47D1	7MF1	RBD	707
B1-182-1	7MLZ	RBD	795
LY-CoV1404	7MMO	RBD	759
PDI-37	7MZF	RBD	1163
PDI-42	7MZG	RBD	1131
PDI-93	7MZJ	RBD	1212
PDI-96	7MZK	RBD	571
PDI-210	7MZL	RBD	1128
PDI-215	7MZM	RBD	1046
PDI-231	7MZN	RBD	1178
WRAIR-2057	7N4I	RBD	839
WRAIR-2173	7N4J	RBD	937
WRAIR-2125	7N4L	RBD	673
Fab2-36	7N5H	RBD	914
ION-360	7NP1	RBD	876
COVOX-222	7NX6	RBD	1025
COVOX-278	7OR9	RBD	841
P5C3	7P40	RBD	414
FD-11A	7PQZ	RBD	781
FD-5D	7PR0	RBD	844
Beta-6	7PRY	RBD	626
Beta-22	7PRZ	RBD	913
Beta-24	7PS0	RBD	761
Beta-27	7PS1	RBD	988
Beta-38	7PS4	RBD	688
Beta-47	7PS5	RBD	826
FI-3A	7Q0G	RBD	957
Beta-49	7Q6E	RBD	944
Beta-50	7Q9F	RBD	954
CV2.1169	7QEZ	RBD	690
CV2.3235	7QF0	RBD	1349
CV2.6264	7QF1	RBD	761
Beta-55	7QNW	RBD	1037
COVOX-58	7QNY	RBD	1344
P2G3	7QTI	RBD	604
C051	7R8N	RBD	941
C548	7R8O	RBD	928
C022	7RKU	RBD	973
C118	7RKV	RBD	1022
PDI-222	7RR0	RBD	861
CC6.33	7RU3	RBD	847
CC6.30	7RU5	RBD	1065
CS23	7S5P	RBD	1084
CS44	7S5Q	RBD	901
CV07-287	7S5R	RBD	631
J08	7S6J	RBD	735
R40-1G8	7SC1	RBD	1115
Liu_10-40	7SD5	RBD	979
Liu_10-28	7SI2	RBD	949
54042-4	7T01	RBD	592
GAR5	7T72	RBD	1083
ADI-62113	7T7B	RBD	874
S2K146	7TAS	RBD	985
A19-61-1	7TB8	RBD	477
DH1042	7THE	RBD	983
002-02	7U0Q	RBD	1084
002-13	7U0X	RBD	853
ADI-55688	7U2E	RBD	657
NE12	7U9O	RBD	874
NA8	7U9P	RBD	1003
CoV11	7URQ	RBD	1161
XG014	7V2A	RBD	869
TAU-2303	7WBZ	RBD	1204
CZ-D7	7WCH	RBD	727
XGv282	7WE7	RBD	735
XGv289	7WE9	RBD	581
XGv347	7WEA	RBD	741
XGv265	7WED	RBD	753
ZB8	7WH8	RBD	1049
6M6	7WJY	RBD	1208
NCV2SG53	7WN2	RBD	906
NCV2SG48	7WNB	RBD	1265
553-15	7WO4	RBD	864
553-60	7WOA	RBD	716
553-49	7WOG	RBD	1138
BD55-3152	7WR8	RBD	895
BD55-4637	7WRJ	RBD	1084
Ab_510A5	7WS4	RBD	748
BD55-3500	7WSC	RBD	923
XGv051	7WTF	RBD	907
XGv264	7WTH	RBD	824
XGv-264	7WTI	RBD	632
XGv286	7WTJ	RBD	558
Ab_55A8	7WWI	RBD	727
ZWD12	7WWL	RBD	696
ZWC6	7WWM	RBD	747
2G1	7X08	RBD	631
UT28K	7X7O	RBD	648
Ab354	7X8W	RBD	743
Ab159	7X8Y	RBD	579
Ab326	7X90	RBD	1011
Ab496	7X91	RBD	981
Ab445	7X92	RBD	1042
ZCB11	7XH8	RBD	804
STS165	7XIC	RBD	1090
P2S-2E9	7XSA	RBD	744
P5S-3B11	7XSB	RBD	749
P5S-2B10	7XSC	RBD	908
BD55-1403	7Y0C	RBD	973
BD55-5549	7Y0V	RBD	827
BD55-5514	7Y0W	RBD	899
FP-12A	7YCK	RBD	881
IS-9A	7YCL	RBD	863
IY-2A	7YCN	RBD	1138
P3E6	7YKJ	RBD	708
XG2v024	7YR1	RBD	872
THSC20.HVTR26	7Z0X	RBD	634
THSC20.HVTR04	7Z0Y	RBD	665
Omi-3	7ZF3	RBD	1008
Omi-25	7ZFD	RBD	806
Omi-42	7ZR7	RBD	666
Omi-38	7ZR8	RBD	945
Pushparaj_Fab47	8A94	RBD	984
BA.2-10	8BBN	RBD	994
BA.2-36	8BBO	RBD	936
BA.2-23	8BCZ	RBD	1113
S728-1157	8D0Z	RBD	1157
AZ090	8DAD	RBD	857
P1D9	8DWA	RBD	964
P2B4	8DXS	RBD	688
GAR12	8DXT	RBD	1035
GAR3	8DXU	RBD	1105
S2X324	8ERQ	RBD	785
Hastie_1C3	8F0G	RBD	602
YB9-258	8HC2	RBD	1186
YB13-292	8HC8	RBD	837
**ADI-77310**	**this study**	**RBD**	**718**
**VYD222**	**this study**	**RBD**	**701**
**Average**			**904**
**Standard deviation**			**151**

REFERENCES

1. S. Elbe, G. Buckland-Merrett, Data, disease and diplomacy: GISAID's innovative contribution to global health. *Glob Chall* **1**, 33-46 (2017).

2. J. Hadfield, C. Megill, S. M. Bell, J. Huddleston, B. Potter, C. Callender, P. Sagulenko, T. Bedford, R. A. Neher, Nextstrain: real-time tracking of pathogen evolution. *Bioinformatics* **34**, 4121-4123 (2018).

3. T. N. Starr, S. K. Zepeda, A. C. Walls, A. J. Greaney, S. Alkhovsky, D. Veesler, J. D. Bloom, ACE2 binding is an ancestral and evolvable trait of sarbecoviruses. *Nature* **603**, 913-918 (2022).

4. V. B. Chen, W. B. Arendall, 3rd, J. J. Headd, D. A. Keedy, R. M. Immormino, G. J. Kapral, L. W. Murray, J. S. Richardson, D. C. Richardson, MolProbity: all-atom structure validation for macromolecular crystallography. *Acta Crystallogr D Biol Crystallogr* **66**, 12-21 (2010).

5. E. Krissinel, K. Henrick, Inference of macromolecular assemblies from crystalline state. *J Mol Biol* **372**, 774-797 (2007).
